# Crystal structure of *cis*-[1,2-bis­(di­phenyl­phosphan­yl)ethene-κ^2^ *P*,*P*′]di­chlorido­platinum(II) chloro­form disolvate: a new polymorph

**DOI:** 10.1107/S2056989018008836

**Published:** 2018-06-21

**Authors:** Jimmy Mugemana, John Bender, Richard J. Staples, Shannon M. Biros

**Affiliations:** a Department of Chemistry, Grand Valley State University, 1 Campus Dr., Allendale, MI 49401, USA; b Center for Crystallographic Research, Department of Chemistry, Michigan State University, East Lansing, MI 48824, USA

**Keywords:** crystal structure, polymorph, *cis*-dppe, C—H⋯Cl hydrogen bonding, face-on C—Cl⋯π inter­actions, offset π–π inter­actions

## Abstract

The title compound, [PtCl_2_(C_26_H_22_P_2_)]·2CHCl_3_ (I), is the third monoclinic polymorph of this platinum(II) complex involving the bidentate ligand *cis*-1,2-bis­(di­phenyl­phosphan­yl)ethyl­ene (*cis*-dppe) [for the others, see: Oberhauser *et al.* (1998*a* ▸). *Inorg. Chim. Acta*, **274**, 143–154, and Oberhauser *et al.* (1995 ▸). *Inorg. Chim. Acta*, **238**, 35–43]. The structure of compound (I) was solved in the space group *P*2_1_/*c*, with one complex mol­ecule in the asymmetric unit along with two solvate chloro­form mol­ecules. The Pt^II^ atom is ligated by two P and two Cl atoms in the equatorial plane and has a perfect square-planar coordination sphere. In the crystal, the complex mol­ecule is linked to the chloro­form solvate mol­ecules by C—H⋯Cl hydrogen bonds and face-on C—Cl⋯π inter­actions. There are also weak offset π–π inter­actions present [inter­centroid distances are 3.770 (6) and 4.096 (6) Å], linking the mol­ecules to form supra­molecular sheets that lie in the *bc* plane.

## Abstract 2

A third monoclinic polymorph of the title compound is described. In the crystal, it exhibits C—H⋯Cl hydrogen bonds and face-on Cl⋯π inter­actions involving the chloro­form disolvate mol­ecules. Inter­molecular weak offset π–π inter­actions are also present between the aromatic rings of the ligands.

## Chemical context   

The rigid compound cis-1,2-bis­(di­phenyl­phosphan­yl)ethyl­ene (cis-dppe) has been widely exploited as a bidentate ligand for transition metals. A selection of recent examples include complexes involving iron(II) (Song *et al.*, 2018 ▸), copper(I) (Trivedi *et al.*, 2017 ▸), gold(I) (Yao & Yam, 2015 ▸), nickel(II) (Schallenberg *et al.*, 2014 ▸), nickel(III) (Hwang *et al.*, 2015 ▸), and palladium(II) and platinum(II) (Song *et al.*, 2017 ▸; Oberhauser *et al.*, 1998*a* ▸). The phospho­rus atoms of this ligand have also been modified to give the corresponding oxide, sulfide and selenide derivatives (Morse *et al.*, 2016 ▸; Duncan & Gallagher, 1981 ▸; Colquhoun *et al.*, 1979 ▸; Aguiar & Daigle, 1964 ▸). Hence, structural studies of the parent bis­phosphine are relevant to a wide array of researchers.
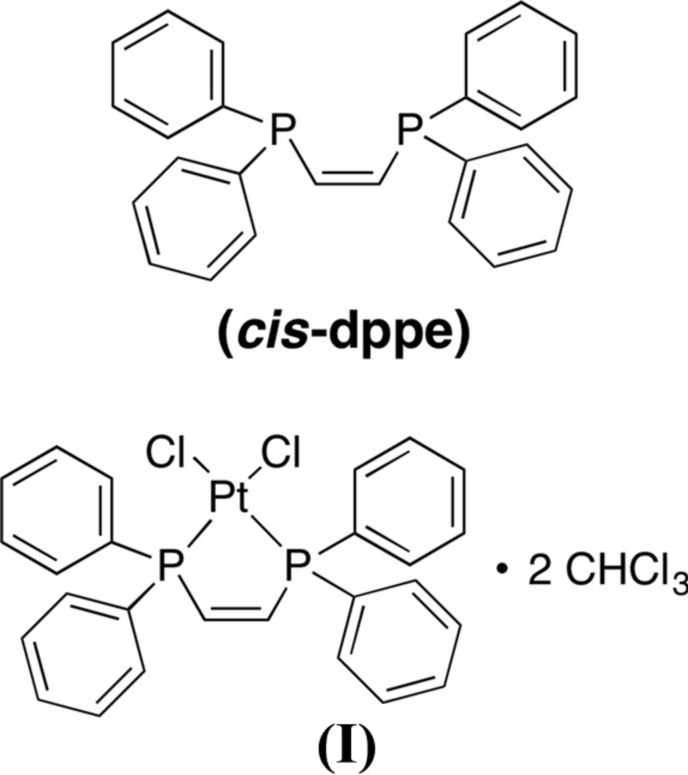


## Structural commentary   

**Figure 1:**
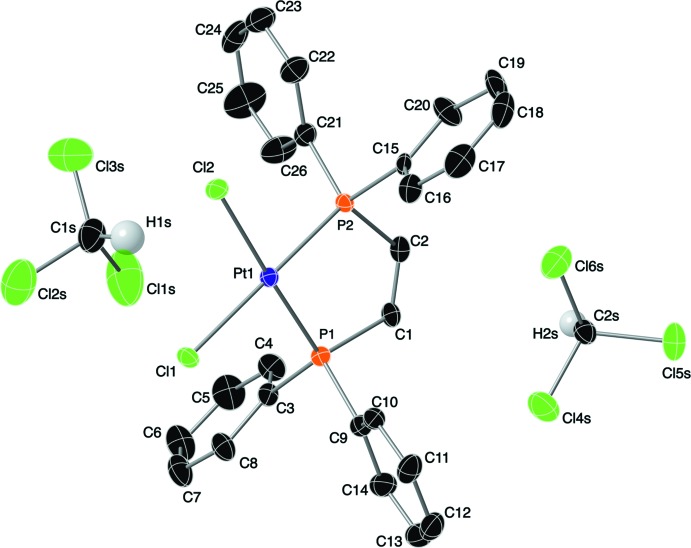
A view of the mol­ecular structure of the title compound, with the atom labeling. Displacement ellipsoids are drawn at the 30% probability level. Hydrogen atoms bonded to the ligand have been omitted for clarity.

The mol­ecular structures of the *cis*-dppe ligand and the title compound (I) are shown in Fig. 1 ▸. This Pt–ligand complex features a square-planar geometry around the Pt^II^ metal center with bidentate coordination by the phospho­rus atoms of the *cis*-dppe ligand. The metal coordination sphere is completed by two chloride anions.

As for the previously reported polymorphs of compound (I), structure HINCIQ (Oberhauser *et al.*, 1998*a* ▸) was solved in space group *P*2_1_/*n* without solvent in the unit cell, while structure ZOLYII (Oberhauser *et al.*, 1995 ▸) was solved in the space group *P*2_1_/*m* as a chloro­form and methyl­ene chloride solvate. The latter complex mol­ecule possesses mirror symmetry with the mirror bis­ecting the Pt atom and central C=C bond of the *cis*-dppe ligand. Selected bond distances and bond angles for the title compound (I), and the two other monoclinic polymorphs are given in Table 1 ▸.

When comparing these two structures to the title compound, the bond lengths and angles around the Pt^II^ center of all three structures are, unsurprisingly, quite similar. The Pt—P bond lengths range from 2.210 (2) to 2.219 (2) Å, while the Pt—Cl bond lengths range from 2.358 (2) to 2.366 (3) Å. The P—Pt—P bond angles range from 86.66 (11) to 87.08 (5)°, while the Cl—Pt—Cl bond angles range from 90.33 (7) to 91.03 (5)°. The τ_4_ descriptor for fourfold coordination (where, for the extreme forms τ_4 =_ 0.00 for square-planar, 1.00 for tetra­hedral and 0.85 for trigonal–pyramidal; Yang *et al.*, 2007 ▸) of the Pt atoms range from 0.02 for compound (I), 0.05 for HINCIQ and 0.0 for ZOLYII, indicating perfect square-planar coordination spheres for each Pt atom.

## Supra­molecular features   

**Figure 2:**
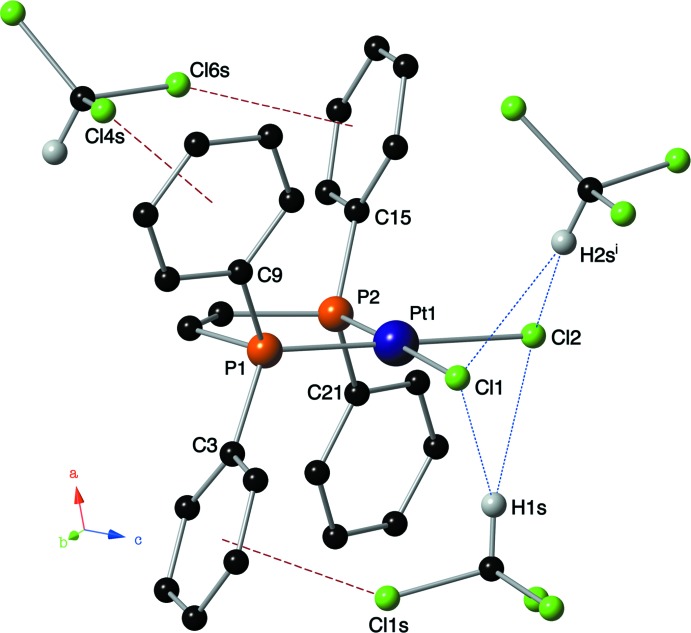
A view along the *b* axis of the title compound showing the C—H⋯Cl hydrogen bonds (blue dotted lines) and chlorine⋯π inter­actions (red dashed lines) found in the crystal lattice [symmetry code: (i) *x*, −*y* + 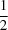, *z* − 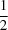].

In the crystal of (I), the metal–ligand complex is linked to the chloro­form solvate mol­ecules by C—H⋯Cl hydrogen bonds and Cl⋯π inter­actions. The hydrogen atoms of both chloro­form mol­ecules are engaged in weak hydrogen bonds with the metal-bound chlorine atoms (Fig. 2 ▸ and Table 2 ▸). The *D*⋯*A* distances range from 3.616 (9) to 3.789 (10) Å, while the *D*—H⋯*A* bond angles range from 132 to 158°. Three face-on Cl⋯π inter­actions (Imai *et al.*, 2008 ▸) are also present involving the chlorine atoms of the chloro­form mol­ecules and the aromatic rings of the *cis*-dppe ligand (Fig. 2 ▸ and Table 3 ▸). The Cl⋯ring centroid distances for these inter­actions range from 3.242 (5) to 3.441 (7) Å, while the C—Cl⋯ring centroid angles range from 139.2 (5) to 160.3 (4)°.

**Figure 3:**
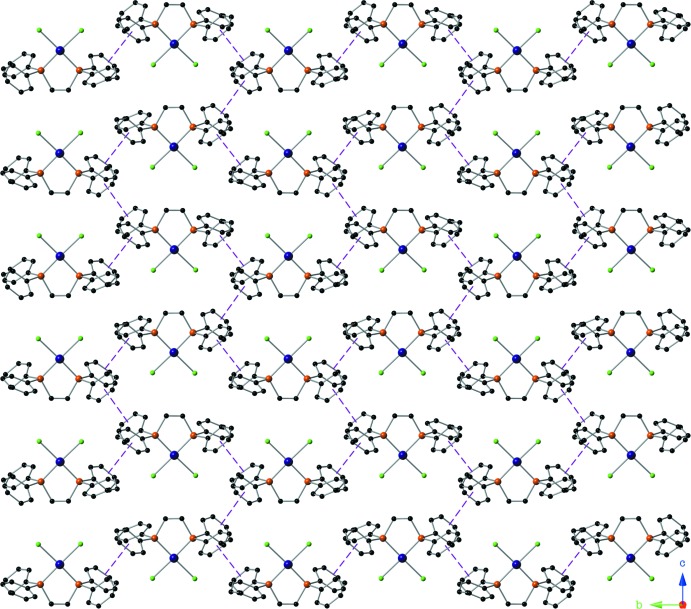
A view along the *a* axis of the weak offset π–π inter­actions (purple dashed lines) between aromatic rings of the title compound, resulting in the formation of supra­molecular sheets. Chloroform solvent molecules have been omitted for clarity.

The complex mol­ecules are also linked by weak offset π–π inter­actions, forming sheets that lie in the *bc* plane, as shown in Fig. 3 ▸. The inter­centroid distances are *Cg*2⋯*Cg*2^ii^ = 4.096 (6) Å [*Cg*2 is the centroid of ring C9–C14, α = 0.0 (5)°, inter­planar distance = 3.917 (4) Å, slippage = 1.20 Å, symmetry code (ii) −*x* + 2, −*y*, −*z* + 1], and *Cg*3⋯*Cg*4^iii^ = 3.770 (6) Å [*Cg*3 and *Cg*4 are the centroids of rings C15–C20 and C21–C26, respectively, α = 5.3 (5)°, inter­planar distances are 3.326 (4) and 3.439 (4) Å, slippage = 1.544 Å, symmetry code (iii) −*x* + 1, *y* − 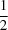, −*z* + 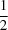].

The closely related polymorph ZOLYII, which contains one CH_2_Cl_2_ solvent mol­ecule and one CHCl_3_ solvent mol­ecule in the unit cell, also shows Cl⋯π inter­actions. However, the methyl­ene chloride solvent mol­ecule is not engaged in a hydrogen bond with a chlorine atom of the Pt^II^ complex, and is disordered in the crystal lattice.

## Database survey   

The Cambridge Structural Database (CSD, version 5.39, February 2018; Groom *et al.*, 2016 ▸) contains 21 structures in which the *cis-*dppe ligand is coordinated to a Pt^II^ center. In addition to the two polymorphs described above, the most similar *cis*-dppe–Pt^II^ coordination complexes include AFEXEO (Vaz *et al.*, 2002 ▸) and FOQPUW (Lobana *et al.*, 2000 ▸), where the Pt^II^ center is bound by two thiol­ate ligands (–SPh and –SPy, respectively). Another structure related to the title compound is KADQEL (Oberhauser *et al.*, 1998*b* ▸) in which the Pt^II^ center is coordinated by two aceto­nitrile mol­ecules. Finally, structure ZOLYOO (Oberhauser *et al.*, 1995 ▸) contains one Pt^II^ center coordinated by two *cis*-dppe ligands with two outer sphere tetra­phenyl­borate mol­ecules as counter-anions. In each of these structures, the bond lengths and angles are similar to those described above for the title compound.

## Synthesis and crystallization   

The title compound was prepared serendipitously by mixing 20.5 mg of *cis*-1,2-dppeSe_2_ (Colquhoun *et al.*, 1979 ▸) with 8 mg of Pt(NCPh)_2_Cl_2_ in CDCl_3_ (0.7 ml) in a NMR tube. This solution was left to stand at room temperature, and colorless needle-like crystals of compound (I) were obtained within a few days.

## Refinement   

Crystal data, data collection and structure refinement details are summarized in Table 4 ▸. The hydrogen atoms were placed in calculated positions and refined as riding: C—H = 0.95–1.00 Å with *U*_iso_(H) = 1.2*U*_eq_(C).

## Supplementary Material

Crystal structure: contains datablock(s) Global, I. DOI: 10.1107/S2056989018008836/su5444sup1.cif

Structure factors: contains datablock(s) I. DOI: 10.1107/S2056989018008836/su5444Isup2.hkl

CCDC reference: 1849747

Additional supporting information:  crystallographic information; 3D view; checkCIF report

## Figures and Tables

**Table 1:** Selected bond distances (Å), bond angles (°) and the fourfold coordination descriptor, τ_4_ *^*a*^*, for the three polymorphs of [(*cis*-dppe)Pt(Cl)_2_]

Compound	(I)	HINCIQ*^*b*^*	ZOLYII*^*c*^*
Pt1—Cl1	2.358 (2)	2.36482)	2.360 (2)
Pt1—Cl2	2.363 (2)	2.366 (3)	2.360 (2)
Pt1—P1	2.217 (2)	2.216 (2)	2.211 (2)
Pt1—P2	2.210 (2)	2.219 (2)	2.211 (2)
P1—Pt1—Cl2	177.58 (7)	176.35 (10)	177.92 (9)
P2—Pt1—Cl1	178.38 (7)	175.81 (10)	177.92 (9)
τ_4_	0.02	0.05	0.0

Notes: (*a*) Yang *et al.* (2007 ▸); (*b*) Oberhauser *et al.* (1998*a* ▸); (*c*) Oberhauser *et al.* (1995 ▸).

**Table 2:** Hydrogen-bond geometry (Å, °)

*D*—H⋯*A*	*D*—H	H⋯*A*	*D*⋯*A*	*D*—H⋯*A*
C1*S*—H1*S*⋯Cl1	1.00	3.04	3.782 (11)	132
C1*S*—H1*S*⋯Cl2	1.00	2.84	3.789 (10)	158
C2*S*—H2*S*⋯Cl1^i^	1.00	2.80	3.616 (9)	139
C2*S*—H2*S*⋯Cl2^i^	1.00	2.77	3.649 (9)	147

Symmetry code: (i) 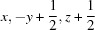.

**Table 3:** Face-on Cl⋯π inter­actions (Å, °) *Cg*1, *Cg*2 and *Cg*3 are the centroids of the phenyl rings C3–C8, C9–C14 and C15–C20, respectively.

C—Cl⋯*Cg*	C—Cl	Cl⋯*Cg*	C⋯*Cg*	C—Cl⋯*Cg*
C1*S*—Cl1*S*⋯*Cg*1	1.706 (11)	3.441 (7)	4.862 (11)	139.2 (5)
C2*S*—Cl4*S*⋯*Cg*2	1.737 (8)	3.242 (5)	4.775 (9)	145.4 (3)
C2*S*—Cl6*S*⋯*Cg*3	1.735 (8)	3.349 (5)	5.017 (9)	160.3 (4)

**Table 4:** Experimental details

Crystal data	
Chemical formula	[PtCl_2_(C_26_H_22_P_2_)]·2CHCl_3_
*M*_r_	901.10
Crystal system, space group	Monoclinic, *P*2_1_/*c*
Temperature (K)	173
*a*, *b*, *c* (Å)	11.1441 (10), 18.0870 (17), 16.9621 (16)
β (°)	106.2465 (10)
*V* (Å^3^)	3282.4 (5)
*Z*	4
Radiation type	Mo *K*α
μ (mm^−1^)	5.04
Crystal size (mm)	0.26 × 0.14 × 0.10

Data collection	
Diffractometer	Bruker APEXII CCD
Absorption correction	Multi-scan (*SADABS*; Bruker, 2013 ▸)
*T*_min_, *T*_max_	0.503, 0.745
No. of measured, independent and observed [*I* > 2σ(*I*)] reflections	26503, 6039, 3360
*R*_int_	0.069
(sin θ/λ)_max_ (Å^−1^)	0.603

Refinement	
*R*[*F* ^2^ > 2σ(*F* ^2^)], *wR*(*F* ^2^), *S*	0.042, 0.122, 1.03
No. of reflections	6039
No. of parameters	352
H-atom treatment	H-atom parameters constrained
Δρ_max_, Δρ_min_ (e Å^−3^)	3.44, −1.01

Computer programs: *APEX2* and *SAINT* (Bruker, 2013 ▸), *SHELXS* (Sheldrick, 2008 ▸), *SHELXL2014* (Sheldrick, 2015 ▸) and *OLEX2* (Dolomanov *et al.*, 2009 ▸; Bourhis *et al.*, 2015 ▸), *CrystalMaker* (Palmer, 2007 ▸).

## supplementary crystallographic information

### Crystal data

**Table:**

[PtCl_2_(C_26_H_22_P_2_)]·2CHCl_3_	*F*(000) = 1744
*M**_r_* = 901.10	*D*_x_ = 1.823 Mg m^−^^3^
Monoclinic, *P*2_1_/*c*	Mo *K*α radiation, λ = 0.71073 Å
*a* = 11.1441 (10) Å	Cell parameters from 6541 reflections
*b* = 18.0870 (17) Å	θ = 2.2–25.4°
*c* = 16.9621 (16) Å	µ = 5.04 mm^−^^1^
β = 106.2465 (10)°	*T* = 173 K
*V* = 3282.4 (5) Å^3^	Needle, colorless
*Z* = 4	0.26 × 0.14 × 0.10 mm

### Data collection

**Table:**

Bruker APEXII CCD diffractometer	3360 reflections with *I* > 2σ(*I*)
φ and ω scans	*R*_int_ = 0.069
Absorption correction: multi-scan (SADABS; Bruker, 2013)	θ_max_ = 25.4°, θ_min_ = 1.7°
*T*_min_ = 0.502, *T*_max_ = 0.745	*h* = −13→13
26503 measured reflections	*k* = −21→21
6039 independent reflections	*l* = −20→19

### Refinement

**Table:**

Refinement on *F*^2^	Primary atom site location: structure-invariant direct methods
Least-squares matrix: full	Secondary atom site location: difference Fourier map
*R*[*F*^2^ > 2σ(*F*^2^)] = 0.042	Hydrogen site location: inferred from neighbouring sites
*wR*(*F*^2^) = 0.122	H-atom parameters constrained
*S* = 1.03	*w* = 1/[σ^2^(*F*_o_^2^) + (0.0396*P*)^2^ + 10.564*P*] where *P* = (*F*_o_^2^ + 2*F*_c_^2^)/3
6039 reflections	(Δ/σ)_max_ = 0.001
352 parameters	Δρ_max_ = 3.44 e Å^−^^3^
0 restraints	Δρ_min_ = −1.00 e Å^−^^3^

### Special details

**Table:**

Geometry. All esds (except the esd in the dihedral angle between two l.s. planes) are estimated using the full covariance matrix. The cell esds are taken into account individually in the estimation of esds in distances, angles and torsion angles; correlations between esds in cell parameters are only used when they are defined by crystal symmetry. An approximate (isotropic) treatment of cell esds is used for estimating esds involving l.s. planes.

### Fractional atomic coordinates and isotropic or equivalent isotropic displacement parameters (Å^2^)

**Table:**

	*x*	*y*	*z*	*U*_iso_*/*U*_eq_	
Pt1	0.76626 (2)	0.25106 (2)	0.26582 (2)	0.02442 (12)	
Cl1	0.75592 (19)	0.15899 (10)	0.16572 (12)	0.0322 (5)	
Cl2	0.7627 (2)	0.34405 (10)	0.16732 (12)	0.0346 (5)	
P1	0.77172 (19)	0.16744 (11)	0.36235 (12)	0.0246 (5)	
P2	0.78048 (19)	0.33570 (10)	0.36196 (12)	0.0243 (5)	
C1	0.7832 (8)	0.2162 (5)	0.4579 (5)	0.0316 (19)	
H1	0.7851	0.1901	0.5068	0.038*	
C2	0.7887 (7)	0.2879 (5)	0.4572 (5)	0.0319 (19)	
H2	0.7970	0.3151	0.5063	0.038*	
C3	0.9055 (8)	0.1058 (4)	0.3861 (5)	0.031 (2)	
C4	1.0070 (9)	0.1237 (5)	0.4509 (6)	0.053 (3)	
H4	1.0036	0.1654	0.4843	0.064*	
C5	1.1147 (11)	0.0803 (6)	0.4674 (7)	0.068 (3)	
H5	1.1841	0.0911	0.5132	0.082*	
C6	1.1195 (11)	0.0210 (6)	0.4160 (6)	0.063 (3)	
H6	1.1943	−0.0068	0.4237	0.075*	
C7	1.0156 (11)	0.0033 (5)	0.3545 (7)	0.059 (3)	
H7	1.0171	−0.0391	0.3217	0.071*	
C8	0.9087 (9)	0.0453 (5)	0.3388 (5)	0.044 (2)	
H8	0.8375	0.0322	0.2952	0.053*	
C9	0.6333 (8)	0.1110 (4)	0.3461 (5)	0.032 (2)	
C10	0.5226 (8)	0.1331 (4)	0.2901 (5)	0.037 (2)	
H10	0.5219	0.1758	0.2575	0.044*	
C11	0.4138 (9)	0.0937 (5)	0.2815 (6)	0.050 (2)	
H11	0.3384	0.1090	0.2430	0.060*	
C12	0.4156 (11)	0.0322 (6)	0.3292 (7)	0.063 (3)	
H12	0.3404	0.0055	0.3241	0.075*	
C13	0.5234 (12)	0.0087 (5)	0.3838 (7)	0.059 (3)	
H13	0.5233	−0.0345	0.4157	0.071*	
C14	0.6331 (10)	0.0481 (5)	0.3925 (6)	0.051 (3)	
H14	0.7084	0.0318	0.4304	0.061*	
C15	0.6507 (8)	0.3979 (4)	0.3499 (5)	0.0290 (19)	
C16	0.5387 (9)	0.3850 (5)	0.2923 (6)	0.046 (2)	
H16	0.5307	0.3446	0.2554	0.055*	
C17	0.4375 (10)	0.4306 (6)	0.2880 (7)	0.066 (3)	
H17	0.3607	0.4217	0.2474	0.079*	
C18	0.4464 (12)	0.4881 (7)	0.3410 (8)	0.066 (4)	
H18	0.3761	0.5188	0.3381	0.079*	
C19	0.5565 (13)	0.5010 (6)	0.3980 (8)	0.069 (4)	
H19	0.5627	0.5414	0.4348	0.083*	
C20	0.6601 (10)	0.4571 (5)	0.4042 (6)	0.054 (3)	
H20	0.7366	0.4670	0.4446	0.065*	
C21	0.9205 (8)	0.3907 (5)	0.3809 (5)	0.035 (2)	
C22	0.9220 (9)	0.4607 (5)	0.3500 (6)	0.051 (3)	
H22	0.8456	0.4828	0.3200	0.061*	
C23	1.0324 (11)	0.4998 (6)	0.3615 (6)	0.060 (3)	
H23	1.0318	0.5488	0.3414	0.072*	
C24	1.1423 (10)	0.4664 (7)	0.4025 (6)	0.061 (3)	
H24	1.2188	0.4922	0.4094	0.073*	
C25	1.1440 (10)	0.3972 (7)	0.4336 (8)	0.085 (4)	
H25	1.2209	0.3755	0.4633	0.101*	
C26	1.0345 (9)	0.3588 (6)	0.4219 (7)	0.062 (3)	
H26	1.0364	0.3097	0.4421	0.074*	
Cl1S	1.1237 (5)	0.1975 (3)	0.2977 (2)	0.1431 (18)	
Cl2S	1.0900 (3)	0.1895 (2)	0.1275 (2)	0.1086 (13)	
Cl3S	1.1475 (6)	0.3253 (3)	0.2052 (5)	0.226 (4)	
C1S	1.0756 (10)	0.2471 (5)	0.2089 (7)	0.066 (3)	
H1S	0.9848	0.2583	0.1995	0.079*	
Cl4S	0.4561 (3)	0.16565 (15)	0.48152 (18)	0.0731 (8)	
Cl5S	0.3791 (3)	0.25490 (16)	0.59773 (16)	0.0679 (8)	
Cl6S	0.4741 (3)	0.32216 (15)	0.47379 (18)	0.0681 (8)	
C2S	0.4841 (8)	0.2468 (4)	0.5386 (5)	0.040 (2)	
H2S	0.5708	0.2447	0.5764	0.048*	

### Atomic displacement parameters (Å^2^)

**Table:**

	*U*^11^	*U*^22^	*U*^33^	*U*^12^	*U*^13^	*U*^23^
Pt1	0.02528 (18)	0.02567 (17)	0.02229 (17)	0.00053 (16)	0.00661 (12)	0.00026 (16)
Cl1	0.0440 (13)	0.0263 (10)	0.0265 (11)	0.0004 (9)	0.0104 (10)	−0.0046 (9)
Cl2	0.0494 (14)	0.0302 (10)	0.0260 (11)	−0.0040 (10)	0.0132 (10)	0.0034 (9)
P1	0.0261 (12)	0.0261 (11)	0.0201 (11)	0.0019 (9)	0.0040 (9)	0.0028 (9)
P2	0.0289 (12)	0.0234 (10)	0.0209 (11)	0.0004 (9)	0.0075 (10)	−0.0012 (9)
C1	0.035 (5)	0.039 (5)	0.022 (5)	0.004 (4)	0.009 (4)	−0.002 (4)
C2	0.026 (5)	0.046 (5)	0.023 (5)	0.004 (4)	0.004 (4)	−0.004 (4)
C3	0.029 (5)	0.031 (4)	0.035 (5)	0.013 (4)	0.011 (4)	0.011 (4)
C4	0.053 (7)	0.050 (6)	0.057 (7)	0.006 (5)	0.016 (6)	−0.002 (5)
C5	0.064 (8)	0.078 (8)	0.056 (7)	0.020 (6)	0.005 (6)	0.013 (6)
C6	0.067 (8)	0.072 (8)	0.046 (7)	0.038 (6)	0.010 (6)	0.013 (6)
C7	0.081 (9)	0.054 (6)	0.051 (7)	0.031 (6)	0.031 (7)	0.011 (5)
C8	0.050 (6)	0.050 (5)	0.030 (5)	0.010 (5)	0.009 (5)	−0.005 (4)
C9	0.032 (5)	0.033 (5)	0.031 (5)	−0.001 (4)	0.008 (4)	0.000 (4)
C10	0.043 (6)	0.036 (5)	0.034 (5)	−0.009 (4)	0.015 (5)	−0.009 (4)
C11	0.036 (6)	0.066 (7)	0.048 (6)	−0.013 (5)	0.014 (5)	−0.004 (5)
C12	0.075 (9)	0.067 (7)	0.059 (7)	−0.035 (7)	0.040 (7)	−0.024 (6)
C13	0.092 (10)	0.040 (6)	0.055 (7)	−0.007 (6)	0.037 (7)	0.001 (5)
C14	0.075 (8)	0.042 (5)	0.039 (6)	−0.010 (5)	0.021 (5)	−0.002 (5)
C15	0.034 (5)	0.029 (4)	0.031 (5)	0.005 (4)	0.021 (4)	0.005 (4)
C16	0.040 (6)	0.057 (6)	0.044 (6)	0.002 (5)	0.015 (5)	0.003 (5)
C17	0.048 (7)	0.070 (8)	0.079 (9)	0.022 (6)	0.019 (6)	0.035 (7)
C18	0.066 (8)	0.071 (8)	0.077 (9)	0.044 (7)	0.046 (7)	0.045 (7)
C19	0.107 (10)	0.048 (6)	0.073 (8)	0.042 (7)	0.058 (8)	0.014 (6)
C20	0.062 (7)	0.045 (6)	0.051 (7)	0.024 (5)	0.010 (6)	−0.002 (5)
C21	0.039 (5)	0.040 (5)	0.026 (5)	−0.006 (4)	0.008 (4)	−0.002 (4)
C22	0.044 (6)	0.055 (6)	0.052 (7)	−0.011 (5)	0.010 (5)	0.002 (5)
C23	0.080 (9)	0.055 (7)	0.046 (7)	−0.026 (6)	0.022 (7)	−0.004 (5)
C24	0.047 (7)	0.089 (9)	0.047 (7)	−0.031 (6)	0.017 (6)	−0.006 (6)
C25	0.034 (6)	0.102 (10)	0.098 (10)	−0.007 (7)	−0.014 (7)	0.019 (8)
C26	0.032 (6)	0.059 (6)	0.085 (8)	−0.005 (5)	0.000 (6)	0.015 (6)
Cl1S	0.165 (4)	0.189 (5)	0.087 (3)	0.079 (4)	0.054 (3)	0.043 (3)
Cl2S	0.075 (2)	0.157 (4)	0.097 (3)	−0.018 (2)	0.029 (2)	−0.011 (3)
Cl3S	0.276 (7)	0.097 (3)	0.395 (10)	−0.060 (4)	0.244 (7)	−0.033 (5)
C1S	0.053 (6)	0.077 (7)	0.075 (8)	0.018 (6)	0.028 (6)	0.019 (7)
Cl4S	0.104 (2)	0.0567 (16)	0.0630 (18)	−0.0037 (16)	0.0315 (17)	−0.0171 (14)
Cl5S	0.0672 (17)	0.095 (2)	0.0518 (16)	0.0031 (17)	0.0334 (14)	−0.0059 (16)
Cl6S	0.0653 (18)	0.0624 (17)	0.080 (2)	0.0084 (14)	0.0262 (16)	0.0302 (15)
C2S	0.044 (5)	0.038 (5)	0.038 (5)	0.009 (5)	0.013 (4)	0.002 (5)

### Geometric parameters (Å, º)

**Table:**

Pt1—Cl1	2.3580 (18)	C12—C13	1.363 (14)
Pt1—Cl2	2.3632 (19)	C13—C14	1.387 (14)
Pt1—P1	2.2173 (19)	C15—C16	1.372 (12)
Pt1—P2	2.2099 (19)	C15—C20	1.397 (11)
P1—C1	1.818 (8)	C16—C17	1.383 (12)
P1—C3	1.814 (8)	C17—C18	1.360 (15)
P1—C9	1.806 (8)	C18—C19	1.353 (16)
P2—C2	1.812 (8)	C19—C20	1.382 (13)
P2—C15	1.798 (8)	C21—C22	1.371 (12)
P2—C21	1.803 (8)	C21—C26	1.392 (12)
C1—C2	1.297 (11)	C22—C23	1.384 (13)
C3—C4	1.377 (12)	C23—C24	1.368 (14)
C3—C8	1.364 (11)	C24—C25	1.355 (15)
C4—C5	1.396 (13)	C25—C26	1.370 (13)
C5—C6	1.393 (14)	Cl1S—C1S	1.706 (11)
C6—C7	1.362 (14)	Cl2S—C1S	1.773 (11)
C7—C8	1.376 (12)	Cl3S—C1S	1.636 (11)
C9—C10	1.388 (11)	Cl4S—C2S	1.737 (8)
C9—C14	1.383 (11)	Cl5S—C2S	1.748 (9)
C10—C11	1.379 (11)	Cl6S—C2S	1.735 (8)
C11—C12	1.373 (13)		
			
Cl1—Pt1—Cl2	90.33 (6)	C14—C9—P1	121.0 (7)
P1—Pt1—Cl1	92.04 (7)	C14—C9—C10	118.9 (8)
P1—Pt1—Cl2	177.58 (7)	C11—C10—C9	120.7 (8)
P2—Pt1—Cl1	178.38 (7)	C12—C11—C10	119.3 (10)
P2—Pt1—Cl2	90.70 (7)	C13—C12—C11	121.2 (10)
P2—Pt1—P1	86.91 (7)	C12—C13—C14	119.7 (10)
C1—P1—Pt1	107.9 (3)	C9—C14—C13	120.2 (10)
C3—P1—Pt1	115.7 (3)	C16—C15—P2	121.2 (7)
C3—P1—C1	104.6 (4)	C16—C15—C20	119.0 (8)
C9—P1—Pt1	115.3 (3)	C20—C15—P2	119.7 (7)
C9—P1—C1	104.8 (4)	C15—C16—C17	120.2 (10)
C9—P1—C3	107.5 (4)	C18—C17—C16	120.9 (11)
C2—P2—Pt1	107.6 (3)	C19—C18—C17	119.2 (10)
C15—P2—Pt1	117.4 (3)	C18—C19—C20	121.8 (11)
C15—P2—C2	103.8 (3)	C19—C20—C15	118.9 (10)
C15—P2—C21	107.7 (4)	C22—C21—P2	123.0 (7)
C21—P2—Pt1	113.2 (3)	C22—C21—C26	117.9 (8)
C21—P2—C2	106.3 (4)	C26—C21—P2	118.9 (7)
C2—C1—P1	117.9 (7)	C21—C22—C23	121.5 (10)
C1—C2—P2	119.6 (7)	C24—C23—C22	118.7 (10)
C4—C3—P1	118.2 (7)	C25—C24—C23	121.2 (10)
C8—C3—P1	121.1 (7)	C24—C25—C26	119.8 (11)
C8—C3—C4	120.5 (8)	C25—C26—C21	120.8 (10)
C3—C4—C5	119.7 (9)	Cl1S—C1S—Cl2S	107.7 (6)
C6—C5—C4	119.3 (11)	Cl3S—C1S—Cl1S	116.9 (8)
C7—C6—C5	119.2 (10)	Cl3S—C1S—Cl2S	109.0 (6)
C6—C7—C8	121.5 (10)	Cl4S—C2S—Cl5S	110.2 (5)
C3—C8—C7	119.5 (9)	Cl6S—C2S—Cl4S	109.9 (5)
C10—C9—P1	119.9 (6)	Cl6S—C2S—Cl5S	111.3 (4)

### Hydrogen-bond geometry (Å, º)

**Table:**

*D*—H···*A*	*D*—H	H···*A*	*D*···*A*	*D*—H···*A*
C1*S*—H1*S*···Cl1	1.00	3.04	3.782 (11)	132
C1*S*—H1*S*···Cl2	1.00	2.84	3.789 (10)	158
C2*S*—H2*S*···Cl1^i^	1.00	2.80	3.616 (9)	139
C2*S*—H2*S*···Cl2^i^	1.00	2.77	3.649 (9)	147

Symmetry code: (i) *x*, −*y*+1/2, *z*+1/2.
